# Potential Impact of Nutrition on Immune System Recovery from Heavy Exertion: A Metabolomics Perspective

**DOI:** 10.3390/nu9050513

**Published:** 2017-05-18

**Authors:** David C. Nieman, Susan Hazels Mitmesser

**Affiliations:** 1 Appalachian State University, North Carolina Research Campus, Kannapolis, NC 28081, USA; 2 Nature’s Bounty Co., Ronkonkoma, NY 11779, USA; susanmitmesser@nbty.com

**Keywords:** immune function, sports nutrition, immunonutrition, immunometabolism, metabolomics

## Abstract

This review describes effective and ineffective immunonutrition support strategies for the athlete, with a focus on the benefits of carbohydrates and polyphenols as determined from metabolomics-based procedures. Athletes experience regular cycles of physiological stress accompanied by transient inflammation, oxidative stress, and immune perturbations, and there are increasing data indicating that these are sensitive to nutritional influences. The most effective nutritional countermeasures, especially when considered from a metabolomics perspective, include acute and chronic increases in dietary carbohydrate and polyphenols. Carbohydrate supplementation reduces post-exercise stress hormone levels, inflammation, and fatty acid mobilization and oxidation. Ingestion of fruits high in carbohydrates, polyphenols, and metabolites effectively supports performance, with added benefits including enhancement of oxidative and anti-viral capacity through fruit metabolites, and increased plasma levels of gut-derived phenolics. Metabolomics and lipidomics data indicate that intensive and prolonged exercise is associated with extensive lipid mobilization and oxidation, including many components of the linoleic acid conversion pathway and related oxidized derivatives called oxylipins. Many of the oxylipins are elevated with increased adiposity, and although low in resting athletes, rise to high levels during recovery. Future targeted lipidomics-based studies will help discover whether *n*-3-polyunsaturated fatty acid (*n*-3-PUFA) supplementation enhances inflammation resolution in athletes post-exercise.

## 1. Background

The immune system is complex, distributed throughout the body, and highly active. Appropriate nutrients are necessary for the varied cells of the immune system to function optimally and respond to injury and invading viruses and bacteria [1,2]. Immune cells need energy fuel substrates (glucose, amino acids, and fatty acids) and multiple nutrients to divide and produce protective chemicals; to move, engulf, and destroy pathogens; and produce proteins (e.g., cytokines and immunoglobulins) and lipid mediators (e.g., prostaglandins, leukotrienes, and specialized pro-resolving mediators). Multiple enzyme systems are involved, with critical roles defined for zinc; iron; copper; selenium; magnesium; vitamins A, B_6_, C, D, and E; and others [1]. Dietary extremes, especially protein-energy malnutrition (PEM), have profound effects in decreasing immune function and increasing risk of opportunistic infections. 

The 2016 Global Nutrition report concluded that one in three people worldwide is malnourished in one form or another [3]. The ultimate goal is to utilize precision nutrition strategies that include an accurate assessment of an individual’s nutritional status and then apply focused nutritional support after considering personal diet, lifestyle, environmental, and genetic information [4]. Although this will take time, there has been increased support for the development of cost-effective, point-of-care devices that can be coupled with smartphones to measure physiologically relevant nutrition biomarkers, which may enable immunonutrition support at an early intervention stage for malnourishment.

Immunonutrition is defined as the use of specific nutritional elements to support and modulate the immune system in a way that benefits a certain condition of physiological stress, disease state, or injury [5]. Immunonutrition support utilizes various formulations and routes of administration. It is recommended for individuals with PEM, patients such as those with cancer, the frail elderly, and during physiologically stressful states resulting from traumatic injuries, major surgeries, and extensive burn injuries [5,6,7]. There is growing interest in immunonutrition support for athletes during heavy training cycles and competitive periods [2,8].

Athletes undergo regular phases of physiological stress, and the immune system reflects this stress with transient decrements in immunosurveillance, increased inflammation, and correspondingly elevated risks of upper respiratory tract infections (URTI) [9,10,11,12]. There is a growing appreciation for high quality nutrition support to mitigate the influence of demanding training schedules on the immune system of athletes [2,13]. This paper will review effective and ineffective immunonutrition support strategies for athletes, with a focus on the benefits of carbohydrates and polyphenols from a metabolomics-based perspective. 

Metabolomics is the study of small molecular weight molecules (metabolites) present in a biological system, and requires sophisticated mass spectrometry platforms, exact quality control procedures, a large reference library of chemical standards, and precise biochemical identification protocols [14]. Metabolomics measurements with gas chromatography–mass spectrometry (GC-MS) began in the 1970s, but was still considered an emerging field as late as 2010, the year some of the earliest exercise-based metabolomics studies with human athletes were published [14,15,16]. Non-targeted and targeted metabolomics-based approaches improve the interpretation of the metabolic response to exercise and nutrition interventions by simultaneously measuring and identifying shifts in hundreds of metabolites from diverse pathways [15,16,17,18,19,20,21,22,23,24,25,26]. The tool of metabolomics can reveal new insights regarding complex metabolic processes following exercise and nutrition interventions, and has the potential to shape a new generation of integrative studies and precision nutrition strategies when combined with appropriate bioinformatics, immunology, molecular epidemiology, genomics, transcriptomics, and proteomics. This review will also emphasize the value of using metabolomics when evaluating the influence of nutrition and exercise on immune function and inflammation. Immunometabolism, a term first used in 2011, is an emerging field of scientific endeavor that merges immunology and metabolism [27]. Interest in immunometabolism is based on the growing realization that nutrition and metabolic disorders such as obesity and diabetes have profound effects on inflammation and immune function. Metabolomics, lipidomics, and proteomics (i.e., multi-omics) are critical methodologies in measuring the multilevel interactions between the metabolic and immune systems. 

### Immune Response to Intensive Exercise, Overreaching, and Overtraining

**Figure 1:**
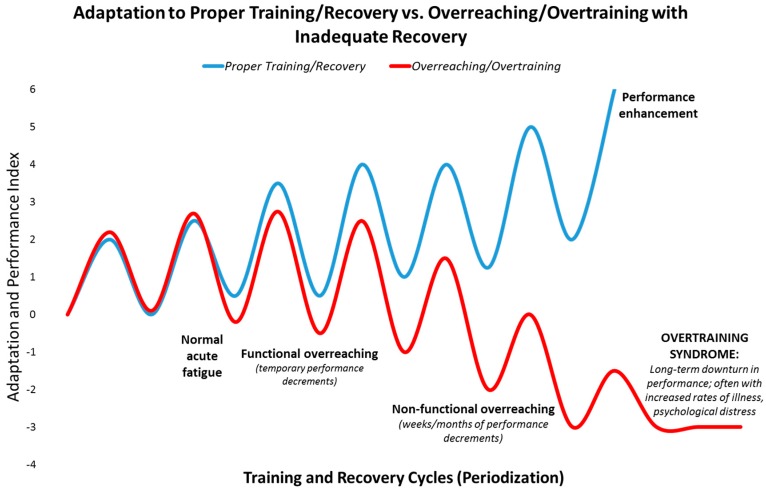
Depiction of the path towards overreaching and the overtraining syndrome compared to performance enhancement.

Athletes seek high level performance by adapting to rigorous training and performance schedules, often intermixed with long distance travel [11,12]. When training and adequate recovery are balanced appropriately, positive physiological adaptations take place (Figure 1). If vigorous exercise workloads are repeated too frequently and exceed the ability to adapt, the athlete can first slip into the zones of “functional and non-functional overreaching” which can be followed in time by the “overtraining syndrome”. This pathway is reinforced when recovery periods and sleep are insufficient, and nutrition support is poor. The consequences are manifold, and include decrements in performance, immune function, and physical and mental health [11,12] (Figure 1). 

Functional overreaching (FOR) occurs during overload periods of training, resulting in a short-term decline in performance and temporary psychological distress. With adequate rest and recovery, individuals going through FOR will eventually experience an improvement in performance. Non-functional overreaching (NFOR) and the overtraining syndrome (OTS) occur when frequency, intensity and/or duration of the training workload exceed the body’s ability to recover, resulting in unusual fatigue, higher rates of illness, mental distress, and inability to perform at expected levels. This process may occur cumulatively over weeks/months through the training cycle and beyond. OTS is characterized by a decrease in expected levels of performance that persists for months and even years despite attempts to recover and scale back training to very low levels [11,12].

The prevalence of NFOR/OTS over the course of an elite athlete’s career is approximately 60%, and about 33% among young athletes and non-elite runners [11,12]. Athletes who have developed NFOR/OTS are at a heightened risk of relapse. Individuals vary widely in their ability to adapt to cycles of training and recovery. Some individuals appear more prone to NFOR/OTS than others exposed to the same period of overload training. Lifestyle factors that can further compound an athlete’s susceptibility to NFOR/OTS include mental stress, sleep problems, inadequate nutrition (e.g., iron deficiency and low carbohydrate diets), and medical disorders. In addition, the symptoms of NFOR and OTS are highly variable between individuals, and can be performance decrements, persistent fatigue, alterations in mood, frequent illnesses (i.e., colds, sore throats, other infections), increased resting and exercise heart rate, loss of appetite and weight loss, irregular menses for female athletes, chronic muscle soreness, sleeping difficulty, loss of libido, irritation or aggression with increased sensitivity to stressors, and loss of competitive drive and desire to train [11,12].

**Figure 2:**
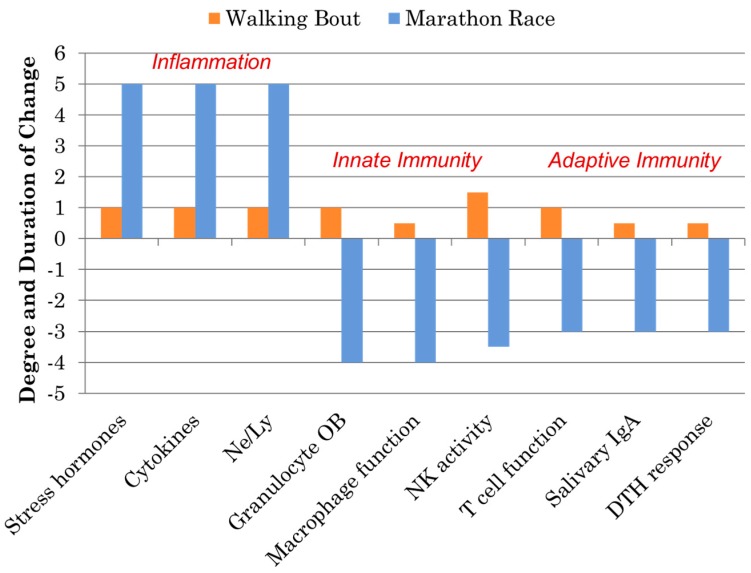
Comparison of the immune responses to a marathon race and a walking bout. (DTH = delayed-type hypersensitivity; NK = natural killer; OB = oxidative burst activity; Ne/Ly = ratio of neutrophil to lymphocyte cell counts, a marker of exercise-induced inflammation).

Athletes participating in one bout of prolonged and intensive exercise such as marathon and ultramarathon race events experience acute physiological stress reflected by muscle microtrauma, oxidative stress, inflammation, and gastrointestinal dysfunction [15,16,17,18,19,20,21,22,23]. Concomitant with these stressors are widespread, transient perturbations in innate and adaptive immunity including decreases in natural killer (NK) cell cytotoxic activity, granulocyte respiratory burst activity, nasal and salivary IgA (sIgA) secretion, delayed-type hypersensitivity (DTH), and mitogen-induced lymphocyte proliferation, as well as extensive alterations in circulating immune cell populations (Figure 2). This period of decreased host protection may last several hours to days depending on the immune measure, and is often followed by elevated URTI rates in the athletes 1–2 weeks after competition. As highlighted in Figure 2, these negative immune responses to marathon-type exertion stand in stark contrast to the favorable improvements in immunosurveillance and reduced illness rates that occur following moderate exercise bouts and training periods involving brisk walking [28,29].

Exercise-induced inflammation involves the secretion of cytokines and chemokines leading to a rapid influx of neutrophils followed by monocytes that differentiate into macrophages. The inflammatory response shifts to a resolution phase that is orchestrated by a switch in lipid mediators from the pro-inflammatory eicosanoids to the specialized pro-resolving mediators (SPMs), inhibition of neutrophil infiltration, and clearance by macrophages [25,26,30]. Metabolomics and lipidomics can be utilized to measure these phases, and will be highlighted later in this review.

## 2. Immunonutrition Strategies

During the 1990s it was discovered that carbohydrate ingestion influenced some aspects of the acute immune and inflammation response to prolonged and intense exercise, which inspired a new line of research in the area of nutrition and exercise immunology [31,32,33]. Nutritional components and products of every conceivable category have been tested for their capacity as countermeasures, with a focus on inflammation and the nonspecific, innate arm of the immune system [2,13,34,35,36,37,38,39,40]. The primary hypothesis is that the risk of immunosuppression and respiratory illness is more effectively countered if the nutritional agent augments functions of post-exercise natural killer cell, macrophage, and granulocyte instead of the slower moving acquired or adaptive immune system [2]. Overall, this strategy of immunonutrition support for physiologically stressed athletes is similar to what is provided to patients recovering from trauma and surgery, the frail elderly, and obese individuals with systemic inflammation and comorbidities [2,5,6,7,8].

Much remains to be studied and discovered (in particular, the countermeasure effect of polyphenols that come in many forms and mixtures) in the fledgling field of nutrition-exercise immunology which dates back to the mid-1990s (see Table 1) [2,13,32,34,40]. As summarized in Table 1, at this time, data from exercise-immune studies are non-supportive or of limited physiological countermeasure significance for the use of various nutritional products including antioxidant vitamins (A, C, and E), minerals, glutamine and other amino acids; herbs such as ginseng and Echinacea; and other similar nutritional components [2,13,40]. Interesting but mixed data have been reported for bovine colostrums, probiotics/prebiotics, β-glucan, fish oil, and vitamin D [13,36,41,42,43,44,45,46,47]. One limitation of these data is the lack of well-designed, long-duration studies applying appropriate outcome measures on large numbers of athletes, especially for supplements that have scientific backing in other populations. For example, vitamin D has an important role in multiple systems that are related to athletic endeavor, i.e., skeletal muscle function, inflammation, and immune function [13]. One study showed that low vitamin D status in endurance athletes was related to increased prevalence of acute respiratory illness and altered immune function during a 16-week winter training period [36]. However, there is not a clear consensus as to what defines vitamin D deficiency and there is a dearth of double-blinded, placebo controlled studies with athletes demonstrating that correcting low vitamin D levels translates to improved immunosurveillance and a lowered risk of URTI [13]. A systematic review and meta-analysis concluded that fish oil (*n-*3-polyunsaturated fatty acids or PUFAs) significantly reduced C-reactive protein (CRP), a systemic inflammation biomarker, compared with controls [48]. Heterogeneity between studies was significant, however, patient cohorts tended to have small sample sizes and short study durations, and *n*-3-PUFA doses in some studies were below the suggested anti-inflammatory threshold of two grams per day. These are common problems in the few studies published evaluating the countermeasure effect of *n*-3-PUFA on post-exercise inflammation in athletes [13].

The proposed benefits of antioxidant supplementation in attenuating oxidative stress and immune dysfunction during exercise remain unsubstantiated, and may work contrary to expectations, as highlighted by the finding that large dose vitamin E supplementation amplified post-race inflammation and oxidative stress in Kona Ironman athletes [2,38]. Glutamine is important for optimal immune function, and a popular rationale is that higher than normal intake is needed to counter exertion-related demands from the immune system. This also applies to many other nutrients. Study results with glutamine supplements are mixed, however, and longer duration studies are needed to determine the optimal dosing regimen and potential immune- and infection- related benefits for athletes during intense training [2,13,35]. 

### Carbohydrates

**Figure 3:**
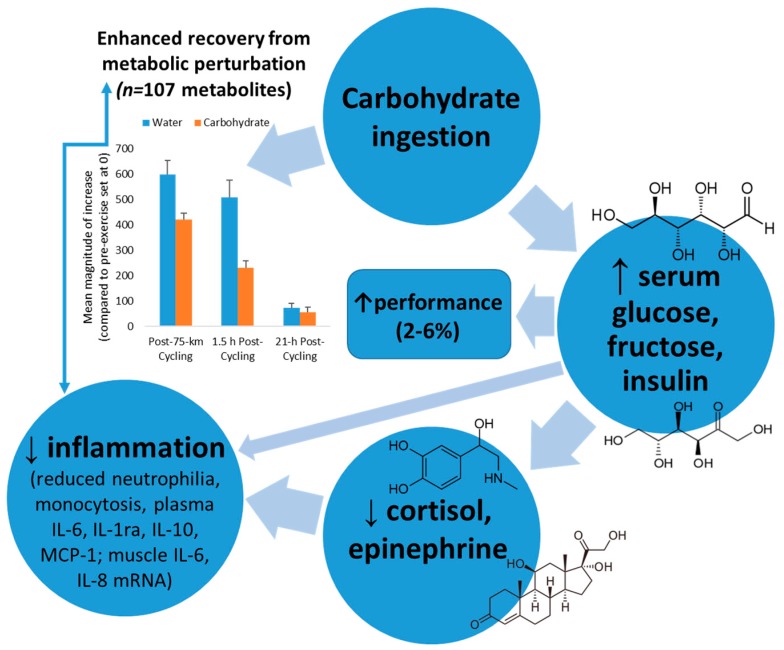
Model linking carbohydrate ingestion with attenuated inflammation and enhanced recovery from metabolic perturbation.

A series of studies dating back to the mid-1990s showed that ingestion of carbohydrate supplements (30–60 grams carbohydrate per hour) during prolonged, intensive exercise attenuated increases in blood neutrophil and monocyte counts, grsanulocyte phagocytosis, stress hormones, and anti-inflammatory cytokines such as IL-6, IL-10, and IL-1ra (Figure 3) [31,32,33,37,49]. At the same time, however, null effects of carbohydrate ingestion were measured for exercise-induced decrements in natural killer cell lytic activity, salivary IgA output, and T lymphocyte proliferative capacity. Thus, carbohydrate ingestion emerged as an effective but partial countermeasure to immune dysfunction during recovery from heavy exertion [2,13,32]. 

Mechanisms through which carbohydrate may exert these impressive countermeasure effects include increasing blood glucose and tissue glucose uptake leading to diminished central nervous system activation and stress hormone output, inhibiting cytokine mRNA expression, lowering beta-oxidation of lipid fuels, reducing pro-inflammatory signals, and attenuating IL-6 release from the working muscle tissue [2,13,32]. Exercising with higher blood glucose levels decreases hypothalamic-pituitary-adrenal activation, leading to moderated release of adrenocorticotrophic hormone and cortisol, growth hormone, and epinephrine (Figure 3). Stress hormones have an intimate link with genes that control cytokine production, and the function of multiple cell types of the immune system. Exercise-carbohydrate interactions, especially during exercise and the early post-exercise recovery period, may modulate signal transduction cascades that influence protein regulatory systems [50,51,52,53].

Thus there is a strong rationale for providing carbohydrate as a countermeasure to exercise-induced inflammation when body carbohydrate stores are challenged. The net result is enhanced recovery from metabolic perturbation, as highlighted in Figure 3. Carbohydrate intake strongly attenuates the magnitude of increase in exercise-induced metabolites from the lipid super pathway, with a quicker recovery rate (Figure 3) [18]. The carbohydrate-induced increase in plasma insulin concentration inhibits adipose tissue triacylglycerol lipase and hormone sensitive lipase, reducing the breakdown of triacylglycerol and decreasing the circulating plasma free fatty acid concentration [18,54]. The net effect is reduced uptake and oxidation of free fatty acids by the contracting muscles, as supported by metabolomics data [18]. Additionally, carbohydrate intake enhances glucose availability to the working muscle resulting in reduced post-exercise stress hormones and inflammation. Studies consistently show an improvement of 2–6% in performance after athletes ingest carbohydrate compared to water during intensive exercise bouts lasting longer than two hours [18,55].

The value of using carbohydrate and other types of immunonutrition support for athletes has been questioned because blocking transient post-exercise elevations in inflammation, oxidative stress, and stress hormones may interfere with important signaling mechanisms for training adaptations [51,52,53]. The literature is limited and not consistent in this area, however, especially in regards to combining carbohydrate intake with heavy training [52]. Training with limited carbohydrate availability may lead to some improved metabolic adaptations, but studies have been unable to link this with performance improvements [52,56]. Part of the problem is that training with limited carbohydrate is difficult, leading to decreased intensity and duration [51]. One argument is that carbohydrate ingestion only partially lowers post-exercise inflammation and stress hormones, allowing sufficient signaling to occur for training adaptations. The value of carbohydrate and other forms of immunonutrition support for athletes during periods of heavy exertion and competitive races should be evaluated by whether or not the athlete has improved recovery, lowered illness rates, reduced muscle damage and soreness, and enhanced overall athletic performance.

#### Polyphenols

Due to their pleiotropic properties and structural diversity, polyphenols have created much interest as potential countermeasures to exercise-induced physiological stress [13,17,18,19,20,24,34,39,40]. The 8000 phenolic compounds are divided into four main classes: flavonoids (Table 2), phenolic acids (e.g., hydroxycinnamic acids as found in coffee, and hydroxybenzoic acids as found in tea and wine), lignans (e.g., as found in whole grains, nuts, and dried fruits), and stilbenes (e.g., resveratrol as found in wine, berries, and grapes) [57]. Flavonoids comprise nearly 50% of all polyphenols, and are divided into simple and complex subclasses as summarized in Table 2 [57]. Improved assessment techniques have led to many recent publications that support a strong and impressive linkage between high dietary polyphenol intake and reduced risks for a wide spectrum of chronic health conditions, overall mortality, acute respiratory illness, and chronic inflammation [58,59,60,61,62]. Flavonoids modulate immunosurveillance outcomes in response to athletic endeavor including natural killer (NK) cell activities, regulatory T (Treg) cell properties, macrophage inflammatory responses, and serum anti-viral effects [63,64]. 

Polyphenols are compounds from plants and are classified as xenobiotics because they are not produced or synthesized by humans. Polyphenols are poorly absorbed in the human small intestine and undergo extensive biotransformation after ingestion [65,66]. A large proportion of ingested plant polyphenols reaches the colon, and microbial degradation produces gut-derived phenolics that can be reabsorbed into the systemic circulation, exert a variety of bioactive effects, and then be excreted in the urine [65,66]. Evidence supports that biological activities of many polyphenols are actually improved following biotransformation [67,68,69]. This process takes time, and hence a prolonged period of increased polyphenol intake is recommended prior to exercise stress interventions to allow body tissues to adapt to the higher phenolic flux level. Although the biotransformed, gut-derived phenolics are suggested to exert anti-inflammatory, anti-viral, anti-oxidative, and other biological effects, these bioactive influences are subtle and, besides using appropriate outcome measures, longer time periods are needed to capture such bioactivities. Supplements in this category that have been investigated include single and combined purified polyphenols (e.g., resveratrol, quercetin), plant extracts (e.g., black currant, bilberry, and green tea), and fruit and vegetable food or juice (e.g., bananas, tart cherry juice, and fresh blueberries). Intervention strategies (dose, duration, frequency, and timing) are still being explored, the optimal polyphenol intake for athletes has not been defined, and intake recommendations have not been established for humans. 

**Figure 4:**
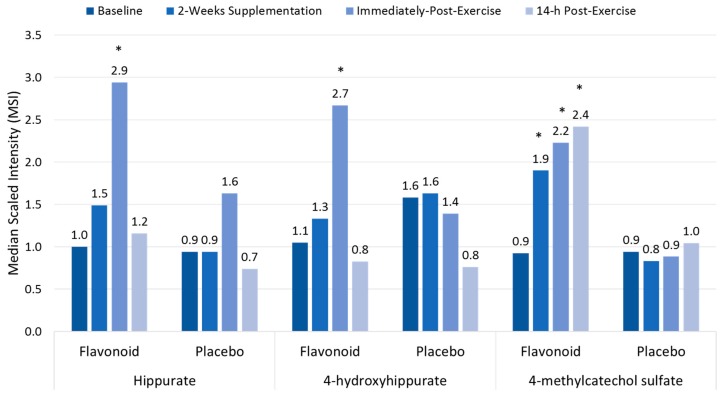
Plasma gut-derived phenolics measured using metabolomics in flavonoid versus placebo groups before and after 14-days supplementation, and immediately and 14-h following a three-day period of intensified exercise. * *p* < 0.05 flavonoid and placebo group contrasts at time point. Data from Reference [17].

Polyphenol-rich plant extracts or supplements have small but significant effects in increasing anti-oxidant capacity, but countermeasure effects on exercise-induced oxidative stress, inflammation, and immune dysfunction using traditional measures have not been consistently measured [13,17,18,19]. Compared to placebo, high blueberry and green tea flavonoid for 17 days (2136 mg/day gallic acid equivalents; equivalent to a daily combined consumption of three whole cups of fresh blueberries and $1\frac{1}{3}$ cups of brewed green tea) was linked to increased plasma levels of gut-derived phenolics and reduced ex vivo viral replication in blood samples from athletes after a three-day overreaching, running protocol [17,63]. As shown in Figure 4, the three-day period of exercise combined with 17 days of flavonoid supplementation augmented the appearance of gut-derived phenolics in circulation, apparently through exercise-induced increases in gut permeability [17]. The athletes exhibited significant inflammation, oxidative stress, and muscle soreness after running at high intensity for 7.5 h during the three-day running period, but no apparent short-term recovery benefit was derived from the elevated gut-derived phenolic signature. 

Additional research is needed to determine if longer-term polyphenol enrichment of the athletic diet mitigates the physiological stress of heavy exertion, improves the speed of recovery, produces other benefits such as lowered incidence of acute respiratory illnesses, and has comparable effects in a variety of athletic groups. The methodology of targeted metabolomics is ideally suited as a methodology to investigate the shifts in gut-derived metabolites following chronic polyphenol supplementation, and human trials are revealing an increasing number of metabolites that appear at high levels in the colon and systemic circulation [22]. 

**Figure 5:**
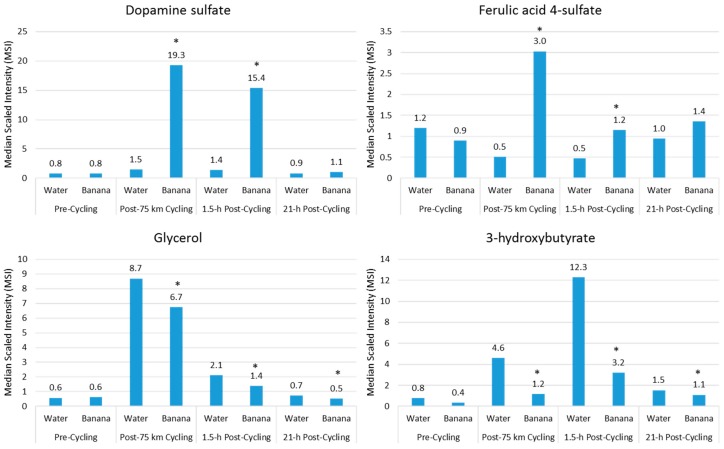
Comparison of banana flesh (dopamine sulfate, ferulic acid 4-sulfate) and lipid metabolites during recovery from 75-km cycling in athletes ingesting water only or water with bananas. (* Data from Reference [18]).

Acute fruit ingestion during exercise also increases plasma levels of many metabolites that are inherent within the flesh of the fruit, and may add to the benefits of fruit sugars in supporting athletic endeavor. For example, compared to water, banana (0.6 g/kg carbohydrate per hour) ingestion before and during 75-km cycling was associated with increased plasma levels of dopamine sulfates and ferulic-acid 4-sulfate, and correspondingly increased post-exercise antioxidant capacity [18] (Figure 5). Banana ingestion was also linked to a meaningful performance enhancement, decreased plasma levels of lipid metabolites (Figure 5), and feelings of improved energy and ability to focus [18]. Several measures of inflammation were attenuated post-exercise in the banana arm, including total leukocytes (23%↓ contrast immediately post-exercise), the neutrophil/lymphocyte ratio (22%↓ 1.5-h post-exercise), plasma IL-10 (56%↓ immediately post-exercise), and serum cortisol (28%↓ 1.5-h post-exercise). Additionally, after banana ingestion, biomarkers associated with carbohydrate availability and oxidation were increased, and these biomarkers include blood glucose and fructose, insulin, and the respiratory exchange ratio. In general, ingestion of fruit flesh sugars and phenolics not only is compatible with high-level exercise performance, but also results in increased plasma levels of fruit-specific metabolites that may provide some protection from oxidative stress, an effect that is best captured using metabolomics [18].

## 3. Metabolomics and Immunometabolism Relationships

Heavy exertion has a profound, acute effect on human metabolism, but most studies have focused on a small, targeted number of biochemical outcome measures. The recent development of metabolomics profiling technologies provides a system-wide view of the metabolic response to exercise, nutrition, and other lifestyle interventions as has been highlighted in this review. Immunometabolism incorporates the metabolite shifts representing the inflammatory response to exercise and nutrition interventions, and will be described in more detail in this section.

**Figure 6:**
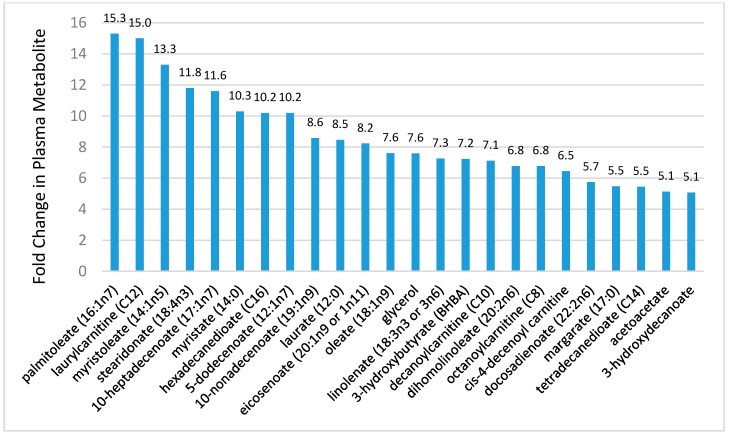
Metabolites increasing ≥5-fold in 24 runners following a treadmill run to exhaustion at 70% VO_2max_. Data from Reference [21].

Although the number of studies is limited, metabolomics-based investigations indicate that prolonged and intensive exercise is associated with shifts in hundreds of metabolites, especially those related to extensive lipid mobilization and oxidation [15,16,17,18,19,20,21,22,23,26,70,71,72,73]. Figure 6 shows 24 metabolites from the lipid super biochemical pathway increased more than five-fold in 24 endurance runners following a treadmill run to exhaustion at 70% VO_2max_ (time and distance to exhaustion, 2.26 ± 0.01 h, 24.9 ± 1.3 km, respectively) [21]. As reported in other studies, the metabolomics analysis revealed exercise-induced changes in 209 metabolites, with a predominance of post-exercise increases in plasma long- and medium-chain fatty acids, fatty acid oxidation products (dicarboxylate and monohydroxy fatty acids, acylcarnitines), and ketone bodies [15,22,23]. Perturbations in many of these metabolites were still apparent in endurance athletes after 14 h of recovery [22]. The relationship between increases in plasma IL-6 and these lipid metabolites was also tested, but no viable statistical model could be established [21]. This is an example of the value of using metabolomics-based procedures in exercise studies, and calls into question the cell culture, rodent-based, and recombinant IL-6 infusion studies indicating that one function for this pleiotropic cytokine is to increase lipolysis and FFA oxidation [74]. 

**Figure 7:**
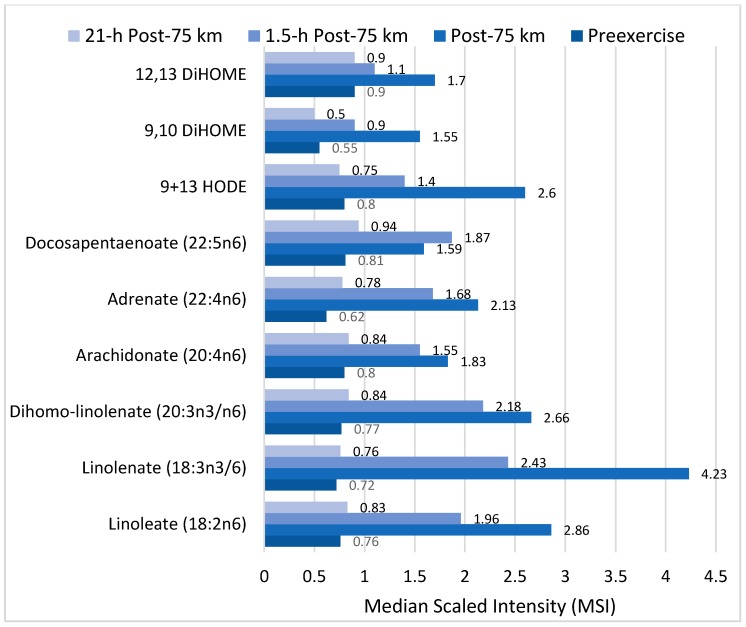
Effect of 75-km cycling on components of the linoleic acid conversion pathway and oxidized linoleic acid derivatives 9+13-HODE, 9,10 DiHOME, and 12,13 DiHOME. Data from Reference [23].

Of particular interest is the effect of exercise on components of the linoleic acid conversion pathway and related oxidized derivatives called oxylipins, many of which are involved in oxidative stress and inflammation responses to intensive and prolonged exercise (Figure 7). Linoleic acid (18:2*n*-6) is an essential fatty acid and the most common polyunsaturated fatty acid (PUFA) in the diet, human plasma and tissue. After ingestion, linoleic acid is converted to longer and more unsaturated fatty acids through enzymatic desaturation and elongation (Figure 7). Linoleic acid is the direct precursor to the stable oxidized metabolite 9- and 13-hydroxy-octadecadienoic acid (9 + 13-HODE) that has been linked to multiple pathological conditions [23,73,75,76]. Plasma 9 + 13-HODE increases strongly following intensive exercise (Figure 6), functions as a biomarker for both oxidative stress and inflammation, and responds to lifestyle interventions such as weight loss [73,75]. The 9 + 13-HODEs have a high plasma concentration compared to other oxlipins, are secreted by a variety of cells including macrophages, endothelial cells, platelets, and smooth muscle cells, and exert biological and signaling activities as proliferator-activated receptor (PPAR) and G protein coupled receptor 132 (G2A) ligands [77,78,79]. Cell injury activates lipoxygenases and may be one pathway through which intensive exercise increases production of HODEs [79]. 

Other direct oxidized derivatives of linoleic acid that increase after intensive, long duration exercise are the isoleukotoxins, (12Z)-9,10-dihydroxyoctadec-12-enoic acid (9,10-DiHome) and (9Z)-12,13-dihydroxyoctadec-9-enoic acid (12,13-DiHOME) (Figure 7) [21,22,23]. 9,10- and 12,13-DiHOMEs are PPAR ligands with potentially wide-ranging effects including adipocyte stimulation and inhibition of osteoblast differentiation. In addition to their role as a PPAR ligand, DiHOMEs exert toxic and oxidative effects, inhibit mitochondrial function, stimulate neutrophil chemotactic activity, and suppress neutrophil respiratory burst activity [80,81].

### Lipid Mediators

Lipidomics is a branch of metabolomics, with a focus on the analysis of lipid components including fatty acids, glycerolipids, glycerophospholipids, sphingolipids, prenol lipids, saccharolipids, and polyketides [25,26,82]. Lipids are essential for biological processes, are important components of signaling pathways, and are closely linked to disease processes. The role of lipid mediators in the pathogenesis of disease, particularly inflammation, is an active, new area of research endeavor [82,83,84,85]. 

**Figure 8:**
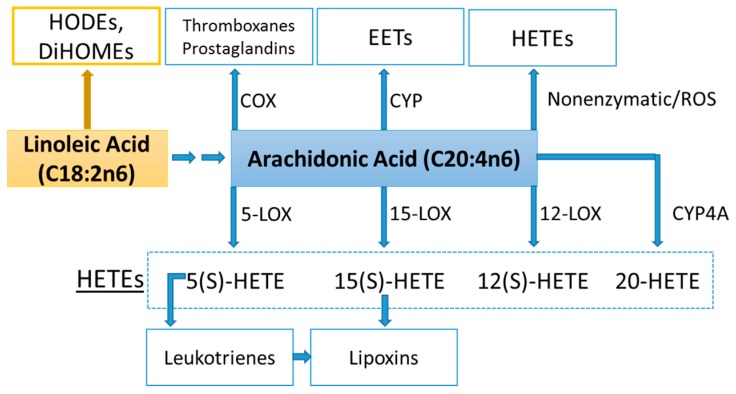
COX, CYP, LOX, and nonenzymatic pathways for biosynthesis of lipid mediators from arachidonic acid. COX = cyclooxygenases; LOX = lipoxygenases; HETEs = hydroxyeicosatetraenoic acids; EETs = epoxyeicosatrienoic acids; CYP = cytochrome P450; CYP4A = cytochrome P450 4A; ROS = reactive oxygen species; HODEs = hydroxyoctadecadienoic acids; DiHOMEs = dihydroxyoctadecenoic acids.

**Figure 9:**
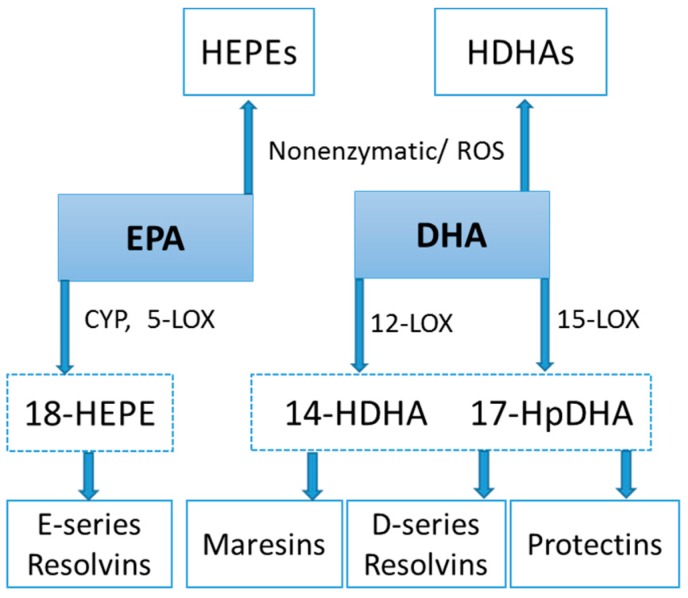
CYP, LOX, and nonenzymatic pathways for biosynthesis of lipid mediators from EPA and DHA.

Lipid mediators are derived from a variety of PUFAs including arachidonic acid (an omega-6 PUFA), and eicosapentaenoic acid (EPA) and docosahexaenoic acid (DHA) (omega-3 PUFAs) (Figure 8 and Figure 9). Most of the PUFA substrate is esterified within cell membrane phospholipids, but can be rapidly released in response to inflammatory or injury stimuli through the enzyme phospholipase A2 (PLA2) [83]. The released PUFAs are changed through both enzymatic and nonenzymatic activities to form oxygenated derivatives called oxylipins [76,83]. Oxylipins can be divided into eicosanoids (synthesized from C20 PUFAs such as arachidonic acid and EPA) and docosanoids (synthesized from C22 PUFAs such as DHA), and also include linoleic acid oxygenated derivatives such as the HODEs. 

Arachidonic acid can be oxygenated by a variety of different enzymes, including lipoxygenases (LOX), cyclooxygenases (COX), and epoxygenases that are catalyzed by cytochrome P450s (CYP), and can be converted to a complex mixture of eicosanoid oxygenated products as a result of lipid peroxidation [76,83]. Figure 8 shows that lipid mediators can also be produced non-enzymatically by autoxidation or by reactive oxygen species (ROS) [84]. Lipidomics measurements using sensitive mass spectrometry platforms allow the simultaneous measurement of large numbers of these lipid mediators, facilitating the systematic investigation of their roles in immune regulation, and inflammation and resolution. 

Prostaglandins and thromboxanes are classic pro-inflammatory initiators, and are produced through the COX pathway (Figure 8). The initial products in LOX pathway reactions are hydroperoxyeicosatetraenoic acids (HpETEs) followed by the more stable hydroxyeicosatetraenoic acids (HETEs) that have multiple roles in the inflammation process [86]. The 5-LOX pathway also produces pro-inflammatory leukotrienes. Leukotriene B4 (LTB4) promotes PMN chemotaxis, and induces the formation of reactive oxygen species and the release of lysosome enzymes by these cells. *n*-3-PUFAs (EPA, DHA) have multiple double bonds and are highly susceptible to oxidation (Figure 9). DHA can be oxidized enzymatically via COX, LOX, or CYP pathways, and nonenzymatically through ROS reactions [76,83]. Both oxidation mechanisms produce a large variety of docosanoid oxidative metabolites. Specialized pro-resolving mediators (SPM) termed resolvins, protectins, and maresins help regulate the resolution phase of acute inflammation [30,87]. Maresins exert anti-inflammatory and pro-resolving responses in macrophages, neutrophils and bronchial epithelial cells and impart beneficial actions in murine models of peritonitis and colitis. Maresin 1 (MaR1) stimulates resolution as well as tissue regeneration, and is produced in macrophages from DHA. 

#### Lipid Mediators, Exercise, Nutrition, and Obesity

During prolonged and intensive exercise, injury to skeletal muscle cells occurs, setting up a well-defined activation of the innate immune system and a corresponding inflammatory response that is acute and self-limited [25,88]. This response involves an initial and rapid infiltration of poly-morphonuclear neutrophils (PMNs) and eosinophils followed by the egress of blood monocytes that differentiate into tissue macrophages. These cells work together with resident tissue cells to promote removal of cell debris and repair. 

This task requires regulated signaling, part of which is supplied by lipid mediators. Initially, pro-inflammatory lipid mediators such as prostaglandins, leukotrienes, and HETES are produced followed by SPMs including resolvins, protectins, maresins, and lipoxins. Although this process is well described in clinical research, few studies have been published regarding the potential roles of lipid mediators in the acute immune and inflammation response to exercise stress [25,26]. 

The first study using metabolomics profiling to measure lipid mediator responses to exercise was published in 2013 by Markworth et al. [26]. Venous blood was collected in 16 healthy male subjects before and after (immediately post-exercise, at 30-min intervals throughout 3 h of recovery, and at 24 h post-exercise) an acute bout of unaccustomed resistance exercise (three sets, 8–10 repetitions, three leg exercises, at loads adjusted to 80% of one repetition maximum). Post-exercise increases were measured for pro-inflammatory prostanoids such as thromboxane B_2_ (TXB2), prostaglandin E_2_ (PGE2), LTB4, several HETEs, and EPA- and DHA-derived resolvins and protectins. The authors surmised that these lipid mediators may have potential roles in immunological and vascular responses to exercise stress including the promotion, regulation, and resolution of PMN and monocyte migration to skeletal muscle [25,26]. 

The author of this review (Nieman, D.C.) conducted a pilot project to provide more information on differences in plasma levels of lipid mediators between overweight/obese individuals and athletes, and the acute increases that can be measured following an intensive 75-km cycling time trial. Participants were 15 overweight/obese females (mean ± SE: age 49.9 ± 1.8 years, BMI 32.9 ± 1.9 kg/m^2^, CRP 4.19 ± 0.8 mg/L) and 3 competitive male cyclists (age 38.7 ± 5.5 years, BMI 28.0 ± 1.9 kg/m^2^, CRP 0.57 ± 0.15 mg/L. Six blood samples were collected from overnight-fasted overweight/obese female subjects from 8:00 a.m. to 12:00 p.m., with the data averaged. A sports drink beverage (8 mL/kg) containing 6% carbohydrate was provided at 10:00 a.m. The three male cyclists reported to the human performance lab in an overnight fasted state, and participated in a 75-km cycling time trial (cycling time, 2.75 ± 0.06 h), while consuming water but no energy from food or beverage. Blood samples were collected pre-exercise, immediately post-exercise, and 1.5-h- and 21-h-post-exercise. Frozen plasma aliquots were sent to the Ambiotis Research Institute (3 Rue des Satellites, 31,400 Toulouse, France) for quantitative, targeted analysis of lipid mediators using liquid chromatography tandem mass spectrometry (LC/MS/MS) [89]. 

**Figure 10:**
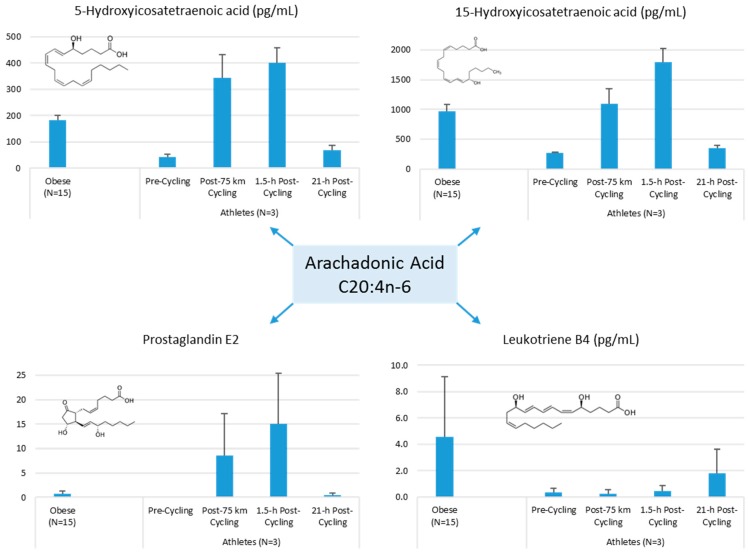
Comparison of selected arachidonic acid lipid mediators in obese subjects and cyclists in the resting state, and acute responses to 75-km cycling.

**Figure 11:**
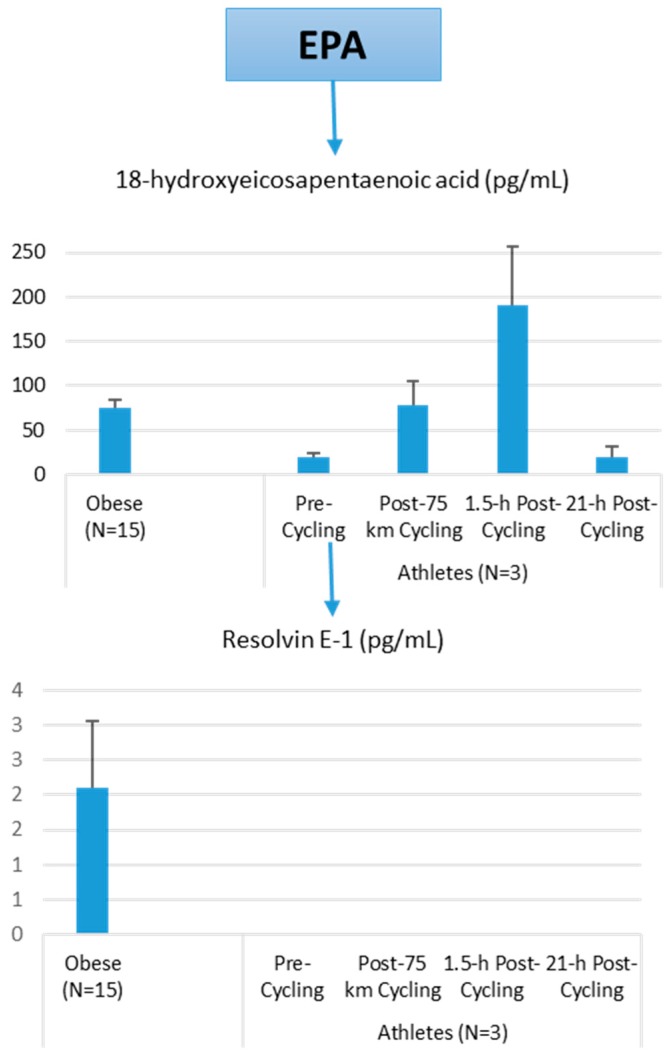
Comparison of selected EPA lipid mediators in obese subjects and cyclists in the resting state, and acute responses to 75-km cycling.

**Figure 12:**
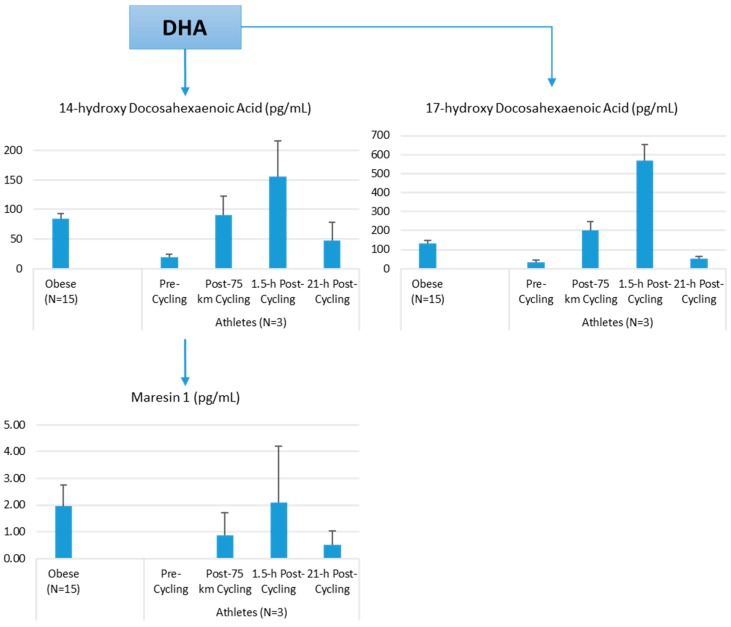
Comparison of selected DHA lipid mediators in obese subjects and cyclists in the resting state, and acute responses to 75-km cycling.

The primary data from this pilot project are summarized in Figure 10, Figure 11 and Figure 12. Most arachidonic acid, DHA, and EPA lipid inflammatory mediator metabolites were substantially lower in the cyclists compared to overweight/obese subjects (resting state). Cycling for 75-km at high intensity caused a transient increase in most lipid inflammatory mediator metabolites that returned close to pre-exercise levels by the next morning (with the exception of LTB4). 12-HETE followed the same pattern as 15-HETE, with a significant difference between overweight/obese and athletic groups in the resting state (76.6 ± 13.6 and 34.4 ± 1.9 pg/mL, respectively). Of the various SPMs, MaR1 (produced in macrophages from DHA) was most responsive to the 75-km cycling bout and thus may be critical for stimulating inflammation resolution and muscle tissue regeneration after intensive endurance exercise. Resolvin E1 (Figure 11) was non-detectable in the endurance athletes before and after exercise, in contrast to elevated levels in the overweight/obese subjects. Lipoxin B4 and protectin D1 were also not detectable in the athletes pre- and post-exercise, but were slightly elevated in the overweight obese subjects (0.68 ± 0.47 and 0.10 ± 0.07 pg/mL, respectively, both *p* = 0.023 compared to zero values in the athletes). Lipoxin A4, and resolvins D1 and D2 were non-detectable in both groups at each time point.

These data demonstrate that adiposity is associated with elevations in plasma levels of many lipid mediators, further characterizing the degree and extent of systemic inflammation using classical biomarkers such as CRP and cytokines [85,90]. Few human studies have been published regarding obesity and oxylipins, but LOX enzymes are active in adipose tissue, acting as upstream regulators of many inflammatory cytokines [91]. Oxylipins signal via cell surface membrane (G protein-coupled receptors) and nuclear receptors (peroxisome proliferator-activated receptors), two pathways playing a pivotal role in adipocyte biology and biochemistry [83]. One human study showed that plasma levels of the pro-inflammatory LOX-metabolites 12-HETE and 5-HETE were significantly reduced following modest weight loss (~7% body mass) in obese males and females with CRP values above 3 mg/L [75]. 

More data are needed on the influence of various dietary patterns on oxylipins [76]. A high *n*-6-PUFA intake (e.g., plant oils) has been linked with a high concentration of *n*-6-PUFA-derived oxylipins, while a high *n*-3-PUFA intake (e.g., fish) has been associated with a high concentration of *n*-3-PUFA-derived oxylipins. As reviewed by Gabbs et al. [76], oxylipins derived from *n*-6-PUFAs generally have greater activity and more inflammatory effects, compared to oxylipins from *n*-3-PUFAs, with notable exceptions. A few supplementation studies indicate that *n*-3-PUFA supplementation is associated with significant changes in the oxylipin profile, but responses vary widely between individuals [92,93,94]. Dietary intake of EPA and DHA leads to increased incorporation of these fatty acids into cell membranes, partly replacing arachidonic acid. Future targeted lipidomics-based studies will help discover whether *n*-3-PUFA supplementation enhances inflammation resolution in athletes post-exercise. Currently, *n*-3-PUFA supplementation studies with athletes are mixed regarding potential effects on post-exercise inflammation and immune function, in part due to weak research designs [13]. Larger and longer duration studies are needed to measure the potential benefits of *n*-3-PUFA supplementation in athletic groups, with more careful consideration given to inflammatory outcomes and the use of targeted lipidomics procedures.

## 4. Conclusions

Athletes experience regular cycles of physiological stress accompanied by transient inflammation, oxidative stress, and immune perturbations. Exercise activates multiple molecular and biochemical pathways, many involving the immune system, and there are increasing data indicating that these are sensitive to nutritional influences. Immunonutrition support has the potential to partially mitigate these exercise-induced changes without interfering with signaling activities that are needed for training adaptations. The most effective nutritional countermeasures include acute and chronic increases in dietary carbohydrate and polyphenols. Other potentially useful nutritional components for athletes include *n*-3-PUFAs, bovine colostrum, probiotics, and β-glucan, but longer term studies with appropriate outcome measures are needed. 

Metabolomics provides a systems-wide approach to this field of scientific endeavor, and has the potential to provide new insights and novel biomarkers with application down to the individual athlete level [95]. Metabolomics is particularly useful in interpreting human responses to nutritional manipulation within the exercise context, and improves the capacity to capture complex and subtle influences on whole body metabolism and physiology. Metabolomics and immunometabolism are challenging, however, with a steep learning curve. Sophisticated bioinformatics support is needed, costs are substantial, and cross-discipline collaboration is required. Nonetheless, this whole-body approach to immunometabolism has the potential to take this field of scientific endeavor to a new level of understanding.

Carbohydrate supplementation has the strongest scientific support, and reduces post-exercise stress hormone levels, inflammation, fatty acid mobilization and oxidation. Ingestion of fruit high in carbohydrates, polyphenols, and metabolites can be just as effective as intake of sports drinks in supporting athletic endeavor, with added benefits including the enhancement of oxidative capacity through fruit metabolites. Additionally, fruit polyphenol intake has the potential for long-term health benefits from increased plasma levels of fruit metabolites and gut-derived phenolics. More research is needed using multi-omics approaches to determine if increased, chronic intake of high carbohydrate-polyphenol food sources has other potential benefits for athletes including enhanced recovery from periodic cycles of hard training and competition, and reduced URTI rates. A high dietary polyphenol intake has been associated in many epidemiological studies with decreased inflammation, oxidative stress, and incidence rates of chronic disease, and investigators are now tasked with discovering through immunometabolism-based studies how these observational data apply to athletic populations. 

Metabolomics and lipidomics data indicate that intensive and prolonged exercise is associated with extensive lipid mobilization and oxidation, including many components in the pathway of linoleic acid conversion and related oxidized derivatives or oxylipins. Many of the oxylipins are elevated with increased adiposity, and although low in resting athletes, rise to high levels during recovery. Oxylipins have an essential role in normal physiology and function, but can also have detrimental effects, especially when systemically elevated because of excessive adiposity. The transient increase in oxylipins following intensive exercise may serve important signaling functions, and metabolomics will help discover whether *n*-3-PUFA supplementation enhances inflammation resolution in athletes post-exercise.

## Figures and Tables

**Table 1:** Summary of immunonutrition supplements, underlying rationale, and recommendations *.

Immunonutrition Supplement	Underlying Rationale	Recommendation Based on Current Evidence
Carbohydrate	Maintains blood glucose during exercise, lowers release of stress hormones; partially counters post-exercise inflammation and related immune changes.	Recommended: 30–70 g per hour of heavy exertion depending on exercise intensity and duration.
High fruit and vegetable intake, with extracts rich in polyphenols	Augment oxidative capacity, anti-viral defenses; gut-derived phenolics aid inherent defenses against long-term inflammation and oxidative stress, improve vascular health, and decrease risk of chronic disease.	Recommended, but the focus should be on long term benefits; extracts reserved for periods of heavy training and competition.
Bovine colostrums	Mix of immune, growth, and hormonal factors in fluid produced by the mammary glands for 24–72 h following calving improves immune function and lower illness risk.	Results are mixed; more data from well-designed studies needed.
Probiotics, prebiotics	Non-pathogenic bacteria in probiotics colonize the gut, improve intestinal microbial flora, and thereby enhance gut and systemic immune function, with a reduction in infection rates; prebiotics provide non-digestible food ingredients that promote the growth of beneficial microorganisms.	Results are mixed; more data from well-designed studies needed.
β-glucan	Receptors on intestinal wall immune cells interact with β-glucan improving innate immunity.	Results are mixed; more data needed comparing different sources.
*n*-3-PUFAs (fish oil) rich in EPA 20:5n3 and DHA 22:6n3	Exert anti-inflammatory and immune-regulatory effects post-exercise; incorporated into cell membranes, partly replacing arachidonic acid and decreasing omega-6-derived mediators.	Results are mixed; data needed with longer duration and improved selection of outcome biomarkers.
Vitamin D	Plays a key role in both innate and acquired immunity through gene expression modulation; athletes may have low vitamin D levels, especially during the winter months.	Results are mixed; data needed on what actually constitutes a deficiency and benefits for correcting low levels.
Glutamine	Important immune cell substrate that may be lowered with prolonged exercise.	Results are mixed; more data from well-designed studies needed.
Vitamin E	Quenches exercise-induced reactive oxygen species (ROS) and augments immunity.	Not recommended; may be pro-oxidative and pro-inflammatory at high doses.
Vitamin C	Quenches ROS and augments immunity.	Not recommended; not consistently different from placebo.
Multiple vitamins and minerals (zinc, magnesium, iron, selenium, manganese)	Work together to quench ROS and reduce inflammation; co-factors for immune responses.	Not recommended; not different from placebo; balanced diet typically sufficient, but may be beneficial if the diet is insufficient. Concerns over blocking ROS signaling for training adaptations.
Amino acids (especially leucine, isoleucine, valine)	Metabolism provides nitrogen for glutamine synthesis.	Not recommended; lack of quality data from controlled studies to recommend amino acid supplementation with exercise.
Herbal supplements (e.g., ginseng, Echinacea)	Contain bioactive molecules that augment immunity and counter infection.	Not recommended; humans studies do not show consistent support within an athletic context.

* Based on References [2,13,31,32,33,34,35,36,37,38,39,40]. “Results are mixed” indicates that results from available studies (in regards to the underlying rationale) are both supportive and non-supportive.

**Table 2:** Flavonoid subclasses and food sources.

Flavonoids	Sample Polyphenols	Food Sources
Simple Flavonoids		
Flavan-3-ols	(+)-catechins, (−)-epicatechin, (−)-epigallocatechin-3-gallate	Green tea, chocolate, tree fruits, grapes, red wine
Flavanones	Hesperetin, Naringenin, Eriodictyol	Citrus fruits and juices
Flavones	Luteolin, Apigenin	Parsley, celery seed, oregano
Isoflavones	Daidzein, Genistein, Glycitein	Soybeans, soy-based foods, legumes
Flavonols	Quercetin, Kaempferol, Myricetin, Isorhamnetin	Onions, apples, tea, berries
Anthocyanidins	Cyanidin, Delphinidin, Malvidin, Pelargonidin, Peonidin, Petunidin	Most berries, stone fruits
**Complex Flavonoids**		
Condensed Tannins (Proanthocyanidins)	Procyanidins, Prodelphinidins, Propelargonidin	Chocolate, stone fruit (apples, pears), grapes, strawberries, cranberries, nut skins, cinnamon, beer, wine, barley, legumes

Source: Reference [57].
